# Using Workshops to Engage Key Stage Three Children in Disposing Food Packaging Sustainably

**DOI:** 10.3390/foods12193542

**Published:** 2023-09-23

**Authors:** Victoria Norton, Niki Alexi, Stella Lignou

**Affiliations:** 1 Sensory Science Centre, Department of Food and Nutritional Sciences, Harry Nursten Building, University of Reading, Whiteknights, Reading RG6 6DZ, UK; victoria.l.norton@reading.ac.uk; 2 Food Quality Perception and Society Team, iSense Lab., Department of Food Science, Faculty of Technical Sciences, Aarhus University, Agro Food Park 48, 8200 Aarhus N, Denmark; niki.alexi@food.au.dk

**Keywords:** children, education, sustainability, food packaging, workshops

## Abstract

Sustainable approaches are generally on the rise; yet clear and accessible information relating to appropriate food packaging disposal is typically lacking. Children need to learn sustainable behaviour from an early age; therefore, targeted education is considered a viable option to inform future generations on sustainable food packaging behaviour. This paper explores children’s behaviour, preferences and knowledge towards food packaging and the role of workshop-based activities in modulating everyday sustainable food packaging behaviour. Two hundred and thirty children (11–14 years old) partook in food packaging workshops involving interactive activities. Children’s most common food packaging issues related to cost, excessive packaging, confusion, motivations, no clear labels, bins being full and no nearby bins. Metal, glass and mixed materials were associated with disposal-related challenges, whereas drinks and fresh produce impacted buying choices from a food packaging perspective. Overall, quiz performance was positive: children were able to identify correctly various food packaging symbols and disposal practices for different food items. In addition, the workshops had a significant impact on learning something new and changing future behaviour. Accordingly, workshops provided an effective approach to engage children in sustainable food packaging behaviour. Future work should focus on strategies to motivate this generation via digital tools to encourage appropriate food packaging behaviour.

## 1. Introduction

Consumers are under increasing pressure to adhere to a more sustainable lifestyle; accordingly, implementing household recycling and adopting appropriate food packaging-disposal practices are considered fundamental starting points [1]. However, cost, lack of interest and standardisation (e.g., uniform packaging), infrastructure challenges and insufficient information can make this surprisingly challenging for consumers [1,2,3,4,5]. For example, in the UK, households recycling is considered complex and highly variable with approximately thirty-nine different bin regimes across the UK, subsequently contributing to littering and incorrect disposal [5]. Moreover, food packaging performs numerous key roles from shelf-life to product information, as well as utilising different material types (such as plastic, glass, metal, paper, cardboard), and poses widespread sustainability-related challenges [6,7,8,9]. Therefore, to encourage sustainable behaviour post-usage a consumer-centric approach is key to helping overcome the differing disposal approaches depending on the specific material [9,10].

Childhood provides an ideal opportunity to develop and learn relevant environmental behaviours, especially between ages seven to fourteen [11]. In addition, children have a noteworthy role in a household behaviour and consume food products; therefore, they need to be able to dispose of packaging appropriately [12,13,14]. It is important that children are well equipped in this area so that they can help to promote sustainable actions, especially since packaging can impact household food wastage (e.g., impacting shelf-life) [13,15]. This could subsequently lead to a positive outcome within a household in terms of sustainable food packaging behaviour (e.g., pester power) [16,17]. Therefore, strategies to encourage such behaviour and implement knowledge in classroom-based settings could be hugely beneficial.

Information that is tailored and targeted could be a viable solution to increasing awareness and knowledge about this hugely important topic, as well as ensuring conscious behaviour [2,7,9,10,18,19,20]. Positively, in this context, education can modulate awareness, knowledge and behaviour while being cost-effective and aiding learning [19,21,22,23,24]. Norton et al. [19] utilised a range of interactive, informative and fun activities focusing on both food packaging symbols and disposal via worksheets and at-home activity booklets; education successfully helped children (key stage two; 7–11 years old) to learn something new and positively influenced their future behaviour. The overall experience was described as positive, and the competition conducted helped to stimulate and inspire creative designs that promoted engagement [19]. In addition, workshops can provide an ideal classroom-based activity and a useful dissemination tool in this area, as well as improve children’s (aged 8–12 years old) recycling knowledge, awareness and intention [22]. Accordingly, it would be useful to test such approaches in an older cohort, for example, key stage three (KS3; children aged 11–14 years old in the first three years of UK secondary school). This age group often undergoes noteworthy development in terms of physical, cognitive, emotional and social behaviours [25]. Therefore, they are a key age group to engage in sustainable practices and will most likely have greater independence in subsequent decision-making compared with younger age groups. 

Children need consistent access to sustainable food packaging information so that they can adopt relevant behaviours; accordingly, emphasis on classroom-based activities could be key to maximising success coupled with tailored approaches. This paper aims to explore KS3 children’s food packaging-related behaviour, knowledge and information preferences via interactive activities. In addition, it examines the effectiveness of age group-specific workshop-based activities in modulating everyday sustainable food packaging behaviour.

## 2. Materials and Methods

### 2.1. Workshops Overview

**Figure 1:**
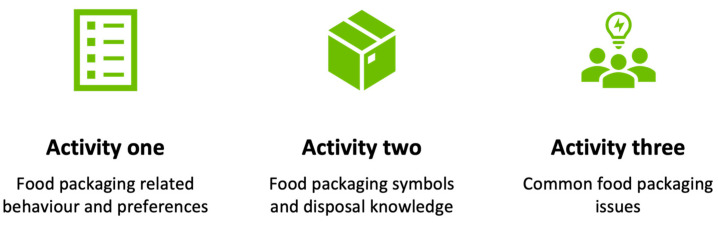
Summary of workshops activities.

Two hundred and thirty children from Reading Secondary Schools (KS3: 11–14 years old, 11.6 ± 0.5 years; 47% male, 50% female and 3.0% other) completed one workshop during school hours in the Spring term (January–March 2023). The workshops were conducted by the project team and focused on how to dispose of food packaging sustainably via three interactive activities such as: (1) an icon-based survey (food packaging-related behaviour and preferences); (2) a multiple choice quiz (food packaging symbols and disposal knowledge); and (3) presentation and discussion (common food packaging-related issues), all designed to promote engagement for this age-group (Figure 1). The workshops received a favourable opinion for conduct from the School of Chemistry, Food and Pharmacy ethics committee (University of Reading; study number 04/2023).

### 2.2. Food Packaging Behaviour and Preferences

**Figure 2:**
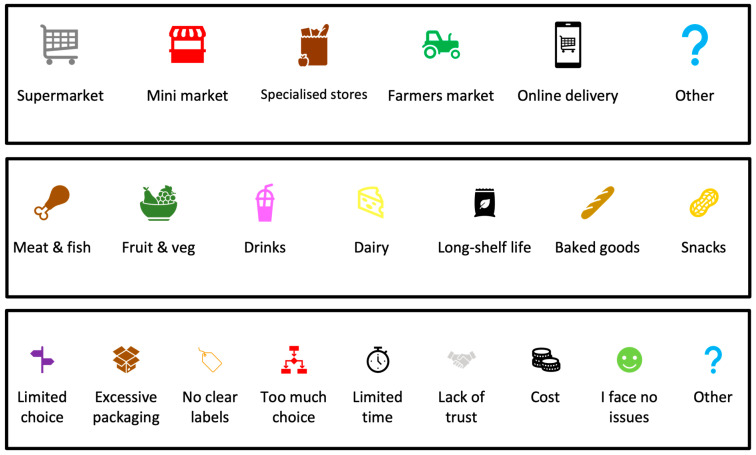
Examples of icons utilised in the food packaging survey.

This activity explored children’s food packaging-related behaviour and preferences using a previously validated survey [10], which was subsequently adapted into an icon-based format to ensure age appropriateness for KS3 children (Figure 2). The survey focused on three sections: (1) the role of food packaging type in product choice (e.g., food shopping decisions, common shopping locations, product choice and food packaging-related issues); (2) food packaging disposal-related challenges (e.g., disposal location issues and food packaging materials); and (3) preferences (e.g., information formats, searching frequency and locations, knowledge gaps and trustworthy sources). The questions were presented in detail on a smart whiteboard, and children were asked to circle the relevant icon in their activity booklet. In addition, the scale types utilised were selected with children in mind, such as single selection (four-to six-point category scales), check-all-that-apply (CATA) and ranking.

### 2.3. Food Packaging Symbols and Disposal Knowledge

**Figure 3:**
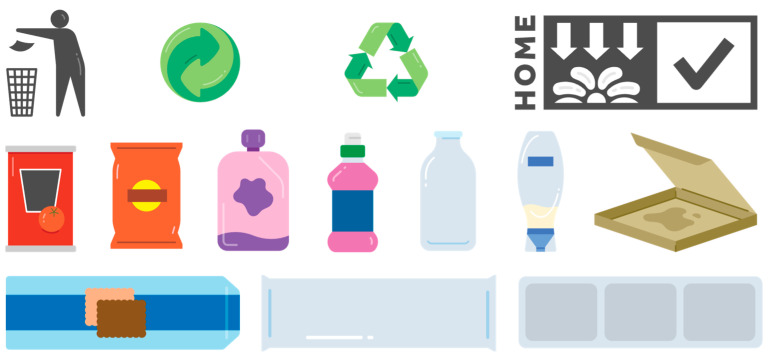
Overview of food packaging symbols and food items used in the quiz.

It is important to understand children’s current knowledge relating to various food packaging symbols and disposal; accordingly, a multiple choice-based quiz was developed based on input from our previous work [19]. The quiz consisted of two sections specifically designed for this age group: (1) symbols (tidyman, green dot, Mobius loop and compost); and (2) appropriate disposal practices for different clean and used food items (fruit juice: clean plastic bottle; tomato soup: clean metal can; milk bottle: clean glass bottle; chocolate biscuits: clean mixed materials (cardboard box, plastic wrapper and plastic tray); mayonnaise bottle: used plastic bottle; pizza box: used cardboard box; yoghurt pouch: used plastic pouch; and crisps wrapper: used plastic wrapper) (Figure 3). The rationale for these questions was to use common UK food packaging symbols and materials (such as plastic, cardboard, mixed materials, metal and glass). Children were asked to determine the correct meaning for each symbol and identify the appropriate bin (general waste, recycling, food waste, bottle bank) for the relevant food item; children completed this task independently in their activity booklet.

### 2.4. Interactive Presentation

The interactive presentation supplemented the activities (one + two), as well as encouraged discussion and enabled opportunities to ask questions. In addition, key topics from our previous work, such as cleaning requirements, how to deal with mixed materials packaging and different bin types in the local Reading area were discussed coupled with additional identified knowledge gaps [19]. Finally, children answered four learning-based questions: (1) do you need to clean food packaging before you dispose of it?; (2) do you need to separate food packaging before you dispose of it?; (3) did you learn something new about disposing of food packaging sustainably?; and (4) do you think you will now change your food packaging-disposal behaviour in the future (all via YES or NO questions)? to understand the impact of education on subsequent behaviour. 

### 2.5. Statistical Analysis

The following statistical analysis was conducted in XLSTAT (version 2022.3.2.1348, New York, NY, USA): (1) Cochran’s Q test was used for check-all-that-apply based questions with multiple comparisons via McNemar’s (Bonferroni) approach; (2) Friedman’s test was utilised for rank-based data using Nemenyi’s procedure for multiple comparisons; and (3) the two-alternative forced choice test (guessing model) was used to identify differences (e.g., YES vs NO or correct vs incorrect responses); *p* < 0.05 was the significance level used for reported data. Data from category scales were expressed in a percentage format and grouped as: (1) disagree (strongly disagree + disagree); (2) neutral (neither agree or disagree); and (3) agree (agree + strongly agree); and (1) at-home (only at-home + more at-home but also on-the-go); (2) equally at-home and on-the-go; and (iii) on-the-go (only on-the-go and more on-the-go but also at-home).

## 3. Results

### 3.1. Food Packaging Behaviour and Preferences

**Figure 4:**
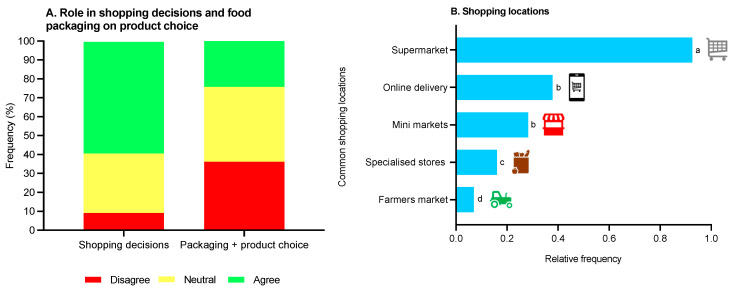
Children’s (n = 230) (**A**) perceived involvement in food shopping decisions and consideration of food packaging type in product choice (disagree: strongly disagree + disagree; neutral: neither agree or disagree; agree: agree + strongly agree; data reported in percentage format); and (**B**) common shopping locations for food products (data expressed as relative frequency and differing letters reflect significance from multiple comparisons).

It was apparent that more than half of the children (59.1%) were involved to some extent in food shopping-based decisions (Figure 4A). However, there was no clear consensus in terms of whether food packaging type had a role in product choice (Figure 4A); the most common food product shopping location was the supermarket (Figure 4B). There was a significant impact (*p* < 0.0001) of food packaging on product choice where children noted that drinks were the most influential, followed by fresh produce (e.g., fruit, vegetables, meat and fish), whereas long-shelf products were least influential (Table 1).

**Figure 5:**
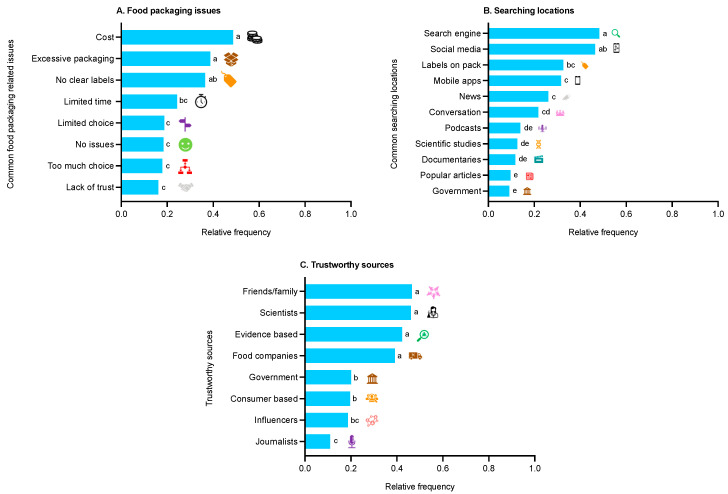
Children’s (n = 230) food packaging: (**A**) overall issues; (**B**) searching locations; and (**C**) trustworthy sources. Data reported as relative frequency (higher values represent more common selections), and differing letters denote significance from relevant multiple comparisons.

It was clear that children’s most common food packaging issues (*p* < 0.0001) were cost and excessive packaging (Figure 5A). However, there was a mixed response in terms of disposal location-related issues, where more problems were found on-the-go (35.2%) or felt they had no issues (35.7%). Children reported significant differences (*p* < 0.0001) in the extent of disposal-related issues for food packaging materials at-home; metal, glass and mixed materials were considered most challenging and paper/cardboard least challenging (Table 1). Children reported key food packaging-related issues (*p* < 0.0001) for at-home as too confusing, no motivation and no clear labels whereas on-the-go as bins being full and no nearby bins (Table 2). 

There were significant differences (*p* < 0.0001) relating to information formats where videos, quizzes and short articles were the most preferred (Table 3). Children were keen to learn more on food packaging topics, such as environmental impact, reusability, recycling and disposal icons (*p* < 0.0001; Table 3). It was apparent that most children (70.9%) were not consciously searching frequently for information on sustainable food packaging behaviour. In addition, children’s key searching locations for more information were related to using search engines and social media (*p* < 0.0001; Figure 5B). There were also significant differences (*p* < 0.0001) in perceived trustworthy sources, where friends/family, scientists, evidence-based organisations and food companies are key (Figure 5C).

### 3.2. Food Packaging Symbols and Disposal Knowledge

**Figure 6:**
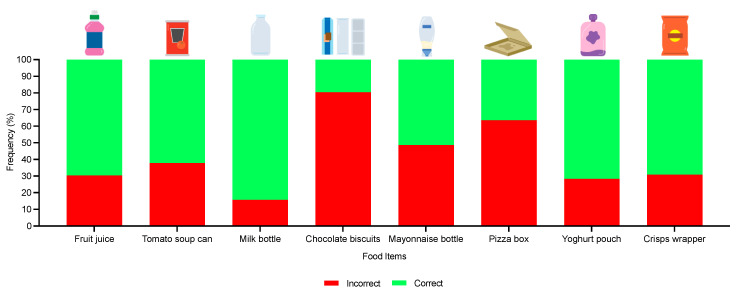
Children’s disposal-related knowledge of various food items (fruit juice: clean plastic bottle; tomato soup: clean metal can; milk bottle: clean glass bottle; chocolate biscuits: clean mixed materials (cardboard box, plastic wrapper and plastic tray); mayonnaise bottle: used plastic bottle; pizza box: used cardboard box; yoghurt pouch: used plastic pouch; and crisps wrapper: used plastic wrapper) and data reported as percentages.

Positively, significantly more children were able to correctly identify the appropriate symbol meaning for the tidyman, Mobius loop and compost symbols; however, this was not the case for the green dot symbol where there was no significant effect (Table 4). There was also a significant effect (*p* < 0.0001) for correct disposal practices for the fruit juice, tomato soup, milk bottle, yoghurt pouch and crisp wrapper, whereas there was no significant difference for the chocolate biscuits (three components: biscuit box, plastic tray and wrapper; *p* = 1.00), mayonnaise bottle (*p* = 0.37) and pizza box (*p* = 1.00) (Figure 6). Overall, individual performance in the quiz was positive as 73% of children answered six or more questions correctly.

### 3.3. Workshops Learnings

**Figure 7:**
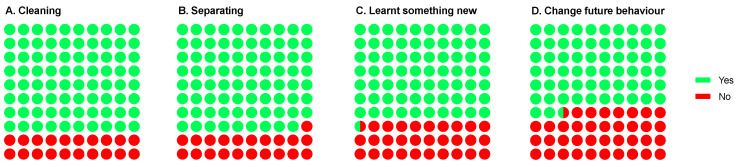
Overview of children’s learning-related questions: (**A**) Do you need to clean food packaging before you dispose of it?; (**B**) Do you need to separate food packaging before you dispose of it?; (**C**) Did you learn something new about disposing of food packaging sustainably?; and (**D**) Do you think you will now change your food packaging-disposal behaviour in the future? Data are reported as percentages.

Children were asked two disposal knowledge-related questions post the interactive presentation and this resulted in a significant outcome (*p* < 0.0001); nearly 80% of childencould correctly identify the need to clean and separate food packaging prior to disposal (Figure 7). In addition, the workshops had a significant impact (*p* < 0.0001) on learning something new (70.4%) and changing future behaviour (62.6%) in this area (Figure 7).

## 4. Discussion

### 4.1. Food Packaging Behaviour and Preferences

It was apparent that most children were involved in food shopping-based decisions; therefore, engaging with this age group in sustainable food packaging behaviour is fundamental. However, there were mixed responses in terms of the role of food packaging type in product choice. This finding was surprising as for older cohorts (18–45 years old) food packaging type impacts product choice [10]. It could be suggested that for this age group (11–14 years old) selecting sustainable packaging is not currently at the forefront of decision making, which is surprising given the growing emphasis on sustainable actions [1]. Hence, it is likely that parents or guardians are still making the majority of decisions and this question may be better suited to older children with even greater independence (e.g., 16–18 years old). As expected, nearly all children purchase food products from the supermarket; accordingly, matching previous work in consumers aged 18–45 years old [10]. Therefore, this implies that supermarkets could be an ideal location to inform households about sustainable practices in a food packaging context; it is estimated that 84% of the UK population shopped at supermarkets in 2022 [26].

Food packaging can contribute to household food wastage; yet this may vary depending on the specific food category [15]. Thus, it is useful to understand which categories children find most challenging from a food packaging perspective; for example, drinks and fresh produce (meat, fish, fruit and vegetables) based products were linked with issues. This might relate to such categories being associated with plastic and relevant environmental concerns, as well as high wastage often resulting from its single-use nature [27,28]. Recently, Norton et al. [10] demonstrated that for UK consumers excessive packaging was a problem in relation to fruit and vegetables; accordingly, a balance between extending shelf life via packaging and food wastage is key going forwards. In addition, food packaging function is considered a key driver of household food wastage in various food categories (e.g., bread, dairy, meat and staples) [15]. For example, Williams et al. [15] highlighted the importance of packaging design: improved size versatility (e.g., to cater for different size households), easy to empty/resealable products and clearer on-pack labelling in minimising household wastage. It should be noted that they found that fruit and vegetables had less packaging-related issues, as they are typically sold unpackaged in Sweden [15]. In terms of food packaging materials, children noted that metal, glass and mixed materials were considered the most challenging for at-home disposal. It could be suggested that metal is associated with canned drink-based consumption and children can be frequent users of such products [29]; therefore, potentially contributing to these findings. It should be noted that in Reading (Berkshire, UK) glass is not kerbside collected; therefore, needs to be taken to a bottle bank which can be considered time consuming [30]. In addition, mixed materials were considered an area that would benefit from addition clarification and education (e.g., improved labelling highlighting different components of packaging often need to be disposed of differently) [19]. Moreover, this would suggest an emphasis on both school and household approaches to maximise opportunities to engage in appropriate disposal practices, as well as tailored information for differing age groups.

Understanding children’s perceived issues relating to food packaging is key to modulating subsequent behaviour. Overall, children noted cost and excessive packaging as common food packaging-related issues. There was no clear consensus on the main location of food packaging-disposal issues; however, common issues related to: (1) at-home (e.g., too confusing, no motivation and no clear labels) and (2) on-the-go (e.g., bins being full and no nearby bins). Moreover, cost and not interest in sustainable practices were also considered two key issues for consumers not adhering to sustainable practices [1]. This suggests that ensuring any changes to food packaging avoid cost implications since price has a key role in purchase-related decisions [3,10,31]. In addition, improving adherence and motivation is key going forwards from an individual and collective perspective [32]. This is especially relevant in a household setting (e.g., at-home) where lack of motivation was more prominent issue compared with on-the-go; accordingly, this may relate to a child’s involvement and engagement levels in the household disposal activities. Infrastructure and lack of standardisation are ongoing issues; this needs to be overcome (via food companies and government involvement) to make it easier for consumers to dispose of food packaging easily regardless of the location [4,5,33].

A key challenge to overcome is encouraging this age group to actively search for relevant information on sustainable practices. Accordingly, it is important to consider children’s preferences so that future activities can be developed to promote interest. It is likely that information presented in videos, quizzes or short article formats on food packaging topics, such as environmental impact, reusability, recycling and disposal icons, could help to encourage engagement with this age group. Positively, videos and podcasts had a noteworthy impact on consumers’ sustainable food packaging behaviours [20,34,35,36]. In addition, children commonly utilised search engines and social media to search for information. These findings reflect the growing digital focus of this generation, as well as the increased access to digital devices and social media compared with previous generations [37]. It could be suggested that future work focus on developing digital campaigns using children’s preferences as a viable approach to reach and engage with this age group.

### 4.2. Food Packaging Symbols and Disposal Knowledge

Children need to be able recognise different food packaging symbols to enable appropriate disposal practices. It was pleasing that children were able to correctly identify three common food packaging symbols (tidyman, Mobius loop and compost); however, less than 31% of the children identified the green dot symbol. In addition, for five of the eight food items, children selected the appropriate disposal patterns, whereas for the remaining three items (e.g., mixed materials and dirty food packaging) this resulted in more incorrect responses. Accordingly, overall, the knowledge level was considered positive; gaps for improvement (such as cleaning and mixed materials) were also noted and this supports previous work in this area [19,23,33]. Improving children’s food packaging-related knowledge (via tailored age-specific education) could help to modulate motivation (which is lacking in this cohort) and thereby assist with overcoming disposal-related barriers; therefore, leading to noteworthy improvements in a household’s daily disposal practices [19,23,33].

### 4.3. Workshops Learnings

Overall, findings were positive and workshops utilising interactive activities (e.g., icon-based surveys, quizzes and presentations) provided an effective approach to engage this age group in disposing of food packaging sustainably. For example, more than half of the children learnt something new and would change future behaviour, which is very encouraging. In addition, at the end of the workshops nearly 80% of children noted that they needed to clean and separate food packaging; therefore, children were able to implement learnings from the discussion points. This supports previous work noting workshops as a tool to communicate findings in a classroom-based setting [22]. Additionally, the type of activity needs to be considered when designing workshops, with worksheets, interactive presentations, competitions and at-home activity booklets also proving influential for children in this area [19]. Going forwards, it is clear that children need regular and consistent access to age-specific sustainable food packaging information, so they can utilise such practices in their everyday lives. It would also be useful to monitor children’s behaviour over a prolonged period with ecological validity to understand the extent of effectiveness and whether engagement fluctuates depending on the specific location (school, at-home or on-the-go). More broadly, children would benefit from food packaging-related issues being addressed within the school curriculum so that they can correctly implement appropriate food packaging disposal from an early age. In addition, expanding the age group and location of the workshops (e.g., across the UK/Europe and different year groups) would be suggested to maximise impact.

## 5. Conclusions

This paper used workshops as an approach to engage KS3 children in disposing of food packaging sustainably. Overall, children’s food packaging-related issues were mainly related to cost, excessive packaging, confusion, motivation, no clear labels, bins being full and no nearby bins. Drinks and fresh produce were key product categories influenced by food packaging and food packaging materials (such as metal, glass and mixed materials) were linked with disposal challenges. Digital platforms (such as videos) could be fundamental in helping this age group to engage in learning and searching for new information. Children’s knowledge relating to food packaging symbols and disposal was positive; however, cleaning and mixed materials were considered associated barriers. The workshops successfully helped the children to learn something new and modulate future behaviour. Going forwards, more emphasis should be placed on a holistic approach, whereby food companies, supermarkets and government are involved in helping schools and households to implement appropriate behaviours. In addition, digital campaigns could be developed using children’s preferences as a viable approach to reach this age group, as well as information tailored for differing age groups to cater for the varying levels of age-related independence. 

## Figures and Tables

**Table 1:** Children’s (n = 230) ranked order relating to the impact of food packaging on product choice and disposal issues at-home for various food packaging materials.

Food Packaging and Product Choice			Disposal Issues and Packaging Materials		
Product Choice	Icon	Mean Ranks	Product Choice	Icon	Mean Ranks
Drinks	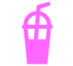	3.34 a	Metal	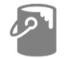	2.80 a
Fresh fruit/vegetables	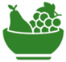	3.61 ab	Glass	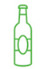	2.92 a
Fresh meat/fish	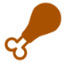	3.75 ab	Mixed materials	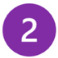	3.00 a
Baked goods	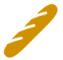	4.00 bc	Hard plastic	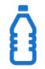	4.43 b
Snacks	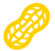	4.05 bc	Soft plastic	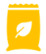	4.61 b
Dairy/non-dairy	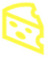	4.37 cd	Bio-based plastic	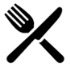	4.83 bc
Long-shelf life	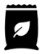	4.88 d	Paper/cardboard	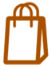	5.41 c

Data reported as mean ranks, where a lower score represents more commonly selected, and different letters denote significance from multiple comparisons.

**Table 2:** Children’s (n = 230) common food packaging-disposal issues at-home and on-the-go.

At-Home			On-the-Go		
Common Issues	Icon	Relative Frequency	Common Issues	Icon	Relative Frequency
Too confusing	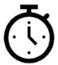	0.45 a	Bins are full	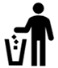	0.57 a
No motivation	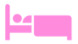	0.44 a	No nearby bin	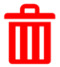	0.53 a
No clear labels	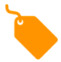	0.33 a	No cleaning	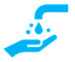	0.24 b
Collection system problems	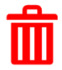	0.20 b	No clear labels	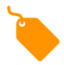	0.20 b
Lack of trust	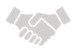	0.10 c	No motivation	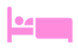	0.19 b
No issues	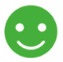	0.08 c	No issues	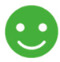	0.16 b

Data expressed as relative frequency and differing letters reflect significance from multiple comparisons.

**Table 3:** Children’s (n = 230) preferred food packaging information formats and topics.

Information Format			Food Packaging Topics		
Formats	Icon	Relative Frequency	Formats	Icon	Relative Frequency
Video	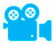	0.69 a	Environment	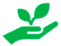	0.50 a
Quiz	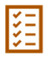	0.31 b	Reusability	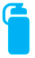	0.40 ab
Short article	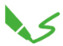	0.28 b	Recycling	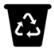	0.40 ab
Interactive chat	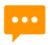	0.28 bc	Disposal icons	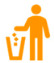	0.37 ab
Audio	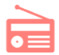	0.23 bc	Reduce packaging	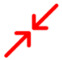	0.36 bc
Infographics	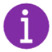	0.17 cd	Packaging materials	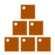	0.30 bc
Long articles	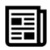	0.10 d	Packaging—why?	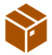	0.24 c

Data are expressed as relative frequency, and differing letters reflect significance from multiple comparisons.

**Table 4:** Children’s (n = 230) food packaging symbol knowledge.

Symbol	Icon	Response (%)		*p*-Value ^1^
		Correct	Incorrect	
Tidyman	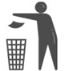	55.7	44.3	0.05
Green dot	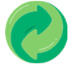	30.9	69.1	1.00
Mobius loop	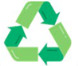	78.7	21.3	<0.0001
Compost	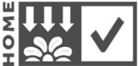	64.8	35.2	<0.0001

^1^*p*-values denote two-alternative forced choice test and data reported as percentages.

## Data Availability

The data presented in this paper are available on request from the corresponding author.

## References

[1] Deloitte. https://www2.deloitte.com/uk/en/pages/consumer-business/articles/sustainable-consumer.html.

[2] Boesen S., Bey N., Niero M. (2019). Environmental sustainability of liquid food packaging: Is there a gap between Danish consumers’ perception and learnings from life cycle assessment?. J. Clean. Prod..

[3] Ketelsen M., Janssen M., Hamm U. (2020). Consumers’ response to environmentally friendly food packaging—A systematic review. J. Clean. Prod..

[4] Burgess M., Holmes H., Sharmina M., Shaver M.P. (2021). The future of UK plastics recycling: One bin to rule them all. Resour. Conserv. Recycl..

[5] Holmes H., Shaver M., Holmes T., Kortsen K. https://www.sustainablefutures.manchester.ac.uk/research/case-studies/one_bin_to_rule_them_all/.

[6] Marsh K., Bugusu B. (2007). Food packaging—Roles, materials, and environmental issues. J. Food Sci..

[7] Otto S., Strenger M., Maier-Noth A., Schmid M. (2021). Food packaging and sustainability—Consumer perception vs. correlated scientific facts: A review. J. Clean. Prod..

[8] Norton V., Waters C., Oloyede O., Lignou S. (2022). Exploring consumers’ understanding and perception of sustainable food packaging in the UK. Foods.

[9] Dornyei K.R., Uysal-Unalan I., Krauter V., Weinrich R., Incarnato L., Karlovits I., Colelli G., Chrysochou P., Fenech M.C., Pettersen M.K. (2023). Sustainable food packaging: An updated definition following a holistic approach. Front. Sustain. Food Syst..

[10] Norton V., Oloyede O.O., Lignou S., Wang Q.J., Vásquez G., Alexi N. (2023). Understanding consumers’ sustainability knowledge and behaviour towards food packaging behaviour to develop tailored consumer-centric engagement campaigns: A Greece and the United Kingdom perspective. J. Clean. Prod..

[11] Otto S., Evans G.W., Moon M.J., Kaiser F.G. (2019). The development of children’s environmental attitude and behaviour. Glob. Environ. Chang..

[12] Castellano G., De Carolis B., D’Errico F., Macchiarulo N., Rossano V. (2021). PeppeRecycle: Improving children’s attitude toward recycling by playing with a social robot. Int. J. Soc. Robot..

[13] Hosany A.R., Hosany S., He H. (2022). Children sustainable behaviour: A review and research agenda. J. Bus. Res..

[14] Ares G., Velazquez A.L., Vidal L., Curutchet M.R., Varela P. (2022). The role of food packaging on children’s diet: Insights for the design of comprehensive regulations to encourage healthier eating habits in childhood and beyond. Food Qual. Prefer..

[15] Williams H., Lindstrom A., Trischler J., Wikstrom F. (2020). Avoiding food becoming waste in households—The role of packaging in consumers’ practices across different food categories. J. Clean. Prod..

[16] Istead L., Shapiro B. (2014). Recognizing the child as knowledgeable other: Intergenerational learning research to consider child-to-adult influence on parent and family eco-knowledge. J. Res. Child. Educ..

[17] O’Neill C., Buckley J. (2019). “Mum, did you just leave that tap running?!” The role of positive pester power in prompting sustainable consumption. Int. J. Consum. Stud..

[18] Patra D., Henley S.C., Benefo E.O., Pradhan A.K., Shirmohammadi A. (2022). Understanding and addressing food waste from confusion in date labelling using a stakeholders’ survey. J. Agric. Food Res..

[19] Norton V., Alexi N., Contente A., Lignou S. (2023). The 3Is: Let’s INVOLVE, INFORM and INSPIRE the next generation on disposing food packaging sustainably. J. Clean. Prod..

[20] Vásquez G., Wang Q.J., Lignou S., Oloyede O.O., Norton V., Alexi N. Impact of co-created digital information campaigns on GenZ and Millennials’ intention to adopt more sustainable packaging behaviours: A Greece and United Kingdom perspective. https://papers.ssrn.com/sol3/papers.cfm?abstract_id=4550271.

[21] Otto S., Pensini P. (2017). Nature-based environmental education of children: Environmental knowledge and connectedness to nature, together, are related to ecological behavior. Glob. Environ. Chang..

[22] Buil P., Roger-Loppacher O., Selvam R.M., Prieto-Sandoval V. (2017). The involvement of future generations in the circular economy paradigm: An empirical analysis on aluminium packaging in recycling in Spain. Sustainability.

[23] Schill M., Godefroit-Winkel D., Hogg M.K. (2020). Young children’s consumer agency: The case of French children recycling. J. Bus. Res..

[24] Trubetskaya A., Scholten P.B.V., Corredig M. (2022). Changes towards more sustainable food packaging legislation and practices. A survey of policy makers and stakeholders in Europe. Food Packag. Shelf Life.

[25] Public Health England. https://www.gov.uk/government/publications/early-adolescence-applying-all-our-health/early-adolescence-applying-all-our-health.

[26] Statista. https://www.statista.com/forecasts/997923/grocery-shopping-by-type-in-the-uk.

[27] Dilkes-Hoffman L.S., Pratt S., Laycock B., Ashworth P., Lant P.A. (2019). Public attitudes towards plastics. Resour. Conserv. Recycl..

[28] White A., Lockyer S. (2020). Removing plastic packaging from fresh produce—What’s the impact?. Nutr. Bull..

[29] Khouja C., Kneale D., Brunton G., Raine G., Stansfield C., Sowden A., Sutcliffe K., Thomas J. (2022). Consumption and effects of caffeinated energy drinks in young people: An overview of systematic reviews and secondary analysis of UK data to inform policy. BMJ Open.

[30] Reading Borough Council. https://www.reading.gov.uk/waste-and-recycling/how-do-i-recycle-in-reading/recycling-banks/.

[31] Oloyede O.O., Lignou S. (2021). Sustainable paper-based packaging: A consumer’s perspective. Foods.

[32] Wang W., Mo T., Wang Y. (2022). Better self and better us: Exploring the individual and collective motivations for China’s Generation Z consumers to reduce plastic pollution. Resour. Conserv. Recycl..

[33] Prestin A., Pearce K.E. (2010). We care a lot: Formative research for a social marketing campaign to promote school-based recycling. Resour. Conserv. Recycl..

[34] Klaiman K., Ortega D.L., Garnache C. (2016). Consumer preferences and demand for packaging material and recyclability. Resour. Conserv. Recycl..

[35] Klaiman K., Ortega D.L., Garnache C. (2017). Perceived barriers to food packaging recycling: Evidence from a choice experiment of US consumers. Food Control.

[36] Wharton C., Vizcaino M., Berardy A., Opejin A. (2021). Waste watchers: A food waste reduction intervention among households in Arizona. Resour. Conserv. Recycl..

[37] Bohnert M., Gracia P. (2021). Emerging digital generations? Impacts of child digital use on metal and socioemotional well-being across two cohorts in Ireland, 2007–2018. Child Indic. Res..

